# Fe-Cr-Mo-B-Si-C Metamorphic Alloy Coating with Excellent Wear Resistance Fabricated via High-Velocity Oxygen Fuel Thermal Spray Process

**DOI:** 10.3390/ma18184241

**Published:** 2025-09-10

**Authors:** Yu-Jin Hwang, Yong-Hoon Cho, Gi-Su Ham, Choongnyun Paul Kim, Kee-Ahn Lee

**Affiliations:** 1 Department of Materials Science and Engineering, Inha University, Incheon 22212, Republic of Korea; yjhwang94@inha.edu (Y.-J.H.); yhcho1539@inha.edu (Y.-H.C.); 2 KOLON Advanced Research Cluster, Kolon Industries Inc., Seoul 07793, Republic of Korea; gisu_ham@kolon.com (G.-S.H.); paulkim@kolon.com (C.P.K.)

**Keywords:** metamorphic alloy, high energy oxygen fuel, coating, wear resistance, microstructure

## Abstract

A cost-effective Fe-Cr-Mo-B-Si-C metamorphic alloy (HXA5) was newly designed and fabricated as coating material using the high-velocity oxygen fuel (HVOF) thermal spray process, and its microstructure and dry wear resistance were investigated in comparison with a conventional HVOF WC-12Co coating. The HXA5 coating material consisted of a splat area and un-melted powder area. The splat area contained metallic glass, (Cr,Fe)_2_B, Cr_2_B, and minor Fe-based BCC phases, and the un-melted powder area was composed of Fe-based BCC, (Cr,Fe)_2_B, and Cr_2_B phases. Room-temperature wear tests revealed that HVOF HXA5 coating material exhibited wear resistance comparable to HVOF WC-12Co coating over ~8.4 km sliding and even superior performance at high-stress wear conditions. This superior wear behavior of HXA5 coating material was attributed to the minimal hardness difference between the metallic glass and boride, the plasticity of the metallic glass, and the formation of a lubricating tribofilm. The wear mechanisms and the influence of alloying elements on glass-forming ability were also discussed.

## 1. Introduction

Thermal spray processing is an effective coating technique widely used in various industrial fields to enhance the service life of materials. In this process, feedstock materials such as powders or wires are typically melted using a high-temperature heat source and then deposited onto the substrate surface by a high-velocity gas stream to form a coating layer. Among thermal spray processes, High-Velocity Oxygen Fuel (HVOF) spraying involves combusting a mixture of oxygen and fuel to generate a supersonic gas flow that carries the powder particles to the substrate, forming the coating. Compared with other thermal spray processes, such as plasma spray and arc spray etc., the HVOF process offers several advantages due to its relatively high particle velocity (approximately 400–800 m/s) and lower processing temperature (approximately 1600–2200 °C), which reduce in-flight oxidation and facilitate the formation of dense, low-porosity coatings [1,2].

Metallic glass alloys are defined as materials in which crystal nucleation is effectively suppressed during the rapid solidification of the molten state, thereby preserving a disordered atomic configuration characterized by short- or medium-range ordering even at ambient temperatures [3]. Owing to the absence of conventional lattice defects, such as dislocations and grain boundaries that are inherent to crystalline metals, metallic glasses exhibit exceptionally high mechanical strength, superior wear resistance, and enhanced corrosion resistance [4]. Thermal spray processes are capable of imparting the ultrafast cooling rates required to induce the formation of metallic glass within the deposited coating material. Thereby thermal spray techniques have attracted significant attention as a promising approach for extending the practical applicability of metallic glasses [5,6]. However, because thermal spray processes primarily utilize high-temperature heat sources to produce coating layers—though it is possible to fabricate coatings containing metallic glass—partial crystallization is often unavoidable, which imposes limitations on suppressing the degradation of the material’s properties [2,7,8,9,10].

Metamorphic alloys were first proposed by Scruggs et al. [11]. These alloys are metal matrix composites (MMCs) composed of crystalline Fe-based phases and metallic borides, which can undergo solid-state amorphization reactions (SSARs) under sustained friction and wear conditions. This unique capability enables the alloys to exhibit excellent wear resistance and prolonged surface protection [12,13,14]. In addition to their superior tribological performance, these alloys are cost-effective due to their primary composition of Fe, Cr, and B, and they can be fabricated using various processing techniques, including thermal spraying, metal injection molding, plasma-transferred arc welding, and spark plasma sintering [14,15,16,17]. In particular, when metamorphic alloys are prepared via thermal spray processes, the rapid solidification resulting from high cooling rates suppresses crystal growth, enabling the in situ formation of metallic glass during deposition. This microstructural feature contributes to the development of coating materials with superior wear resistance [12,13,14,18,19,20]. Research on metamorphic alloys has primarily focused on further enhancing their wear resistance. Jin et al. [21] reported that, after fabricating the material using a detonation gun coating process and conducting wear tests, the coating exhibited wear resistance approximately 45 times higher than that of the substrate. Lee et al. [22] fabricated a metamorphic alloy using a high-energy electron beam irradiation process, where the resulting alloy contained up to 64% boride phase and demonstrated hardness and wear resistance that were two to four times greater than those of the substrate. Sorour et al. [16] fabricated a metamorphic alloy using the spark plasma sintering process. Although amorphization did not occur due to the relatively slow cooling rate of the process, the strengthening effect of the precipitates reduced the wear rate by 80% (higher wear resistance) compared with that of carbon steel. In the previous work, the authors developed a novel Fe-Cr-B-based metamorphic alloy optimized for thermal spray processing by adding Nb and Mo to enhance its glass-forming ability (GFA), which effectively improved the wear resistance of the resulting metamorphic alloy coatings [18,20]. However, the Nb utilized in the alloy developed by the present research group is relatively expensive, which imposes limitations on improving its economic feasibility. Furthermore, for the practical application of metamorphic alloys, it is essential to evaluate their long-distance wear behavior and to conduct comparative studies against representative wear-resistant materials, such as conventional WC-based MMCs. Nevertheless, research in this area remains very limited.

In this study, a cost-effective modified metamorphic alloy was designed by eliminating the expensive alloying element Nb, while compensating for the consequent reduction in glass-forming ability (GFA) through the increased incorporation of B and C. The alloy was deposited as a coating layer via the high-velocity oxygen fuel (HVOF) thermal spray process, and its microstructural characteristics and long-distance wear performance were investigated. Furthermore, the wear mechanism of the newly developed coating was elucidated from a microstructural perspective. For benchmarking purposes, a HVOF-sprayed WC-12Co coating, a widely used representative wear-resistant material, was also fabricated, and its properties were compared to check the practical industrial applicability of the proposed metamorphic alloy coating.

## 2. Experimental Methods

### 2.1. Materials Preparation

In this study, Fe-Cr-B-based metamorphic alloy powders with a modified composition (hereafter referred to as HXA5) were fabricated via the vacuum induction melting inert gas atomization (VIGA) process, and its chemical composition is listed in Table 1. Coatings were fabricated using the HVOF process (TAFA JP-8000, Praxair, Danbury, CT, USA) and the processing parameters employed in this study are summarized in Table 2. SCM440 steel was used as the substrate for both the HXA5 and WC-12Co coatings.

The specimens for cross-sectional microstructural observation were prepared by sequential grinding with SiC papers (from P800 to P4000), followed by polishing with diamond suspensions (from 1 μm to 0.05 μm). All grinding and polishing procedures were performed using an automatic grinding/polishing system (Metprep PH-3, Allied High-Tech Products, Cerritos, CA, USA) under the following parameters: an applied force of 4–22 N, a rotation speed of 80–150 rpm, and a complementary grinding direction.

### 2.2. Characterization and Testing Methods

The size distribution and average particle size of the initial powders were characterized using a laser diffraction particle size analyzer (Mastersizer 3000, Malvern PANalytical, Malvern, UK). The phase constituents of the initial powders and fabricated coatings were identified via X-ray diffraction (XRD, X’Pert Pro MRD, Malvern PANalytical, Malvern, UK) using a Cu Kα radiation source under the following conditions: 2θ scanning range of 20–90°, step size of 0.05°, and scan rate of 1°/min. The cross-sectional microstructure and powder morphologies were examined using a field-emission scanning electron microscope (FE-SEM, MIRA3 XMH, Tescan, Brno, Czech Republic). An electron backscatter diffraction (EBSD, Symmetry, Oxford Instruments, Abingdon, UK) analysis was also performed to evaluate phase distribution. The nanoscale microstructure was examined using a field-emission transmission electron microscope (FE-TEM, JEM-2200FS, JEOL, Tokyo, Japan), and chemical compositions were characterized via electron energy loss spectroscopy (EELS) with the DigitalMicrograph 3.1.1 software (Gatan Inc., Pleasanton, CA, USA). The porosity of the coatings was evaluated by observing the microstructure using an optical microscope (BX-53M, Olympus, Tokyo, Japan), and the average porosity was calculated with an image analysis program (Image-Pro Plus v.4.5.0.29). To determine the volume fractions of the metallic glass and crystalline regions in the metamorphic alloy, chemical etching was performed using an etchant composed of 10 mL of HNO_3_, 3 mL of HCl, 10 g of FeCl_2_, and 77 mL of ethanol. The thermal behavior of the fabricated coating layers was examined by differential scanning calorimetry (DSC; STA449 FE, NETZSCH, Selb, Germany) under an Ar atmosphere, with heating conducted from room temperature to 1300 °C at a rate of 10 °C/min.

The hardness of the coating layers was measured using a micro-Vickers hardness tester (HM-200, Mitutoyo, Kawasaki, Japan). Since thermally sprayed coatings may exhibit variations in hardness between the splat areas and the un-melted powder areas, measurements were performed under a low load of 0.3 kgf and repeated more than 20 times to ensure reliability. The dry wear test was conducted using a pin-on-disk tribometer (RB-102PD, R&B, Daejeon, Republic of Korea) in accordance with ASTM G99 standards [23]. Round tip pure Al_2_O_3_ pins (purity: ≥99.6%, curvature radius: 3mm; Ø 5 × 25 mm; R_a_ = 3.2 μm; manufactured by Dandan Co., Ltd., Daejeon, Republic of Korea) were employed as the counter material. All tests were performed at room temperature (laboratory environment, 20–25 °C). The detailed testing parameters were as follows: applied loads of 29 N, 39 N, and 49 N; a sliding speed of 0.39 m/s; and a total duration of 6 h. Each wear test was repeated twice to ensure reproducibility. After the tests, the wear volume was determined using a confocal laser microscope (VR-6000, Keyence, Osaka, Japan), and the wear rate was calculated according to Equation (1).

(1) $$wear\;rate=\frac{V}{D\times F}$$

Here, *V* represents the volume loss (mm^3^), *D* is the sliding distance (m), and *F* is the applied load (N). After the wear tests, the worn surfaces of the samples were examined using FE-SEM coupled with energy-dispersive X-ray spectroscopy (EDS, X-Max 50, Oxford instruments, Abringdon, UK).

## 3. Results

### 3.1. Initial Powder Feedstock Analysis Results

**Figure 1:**
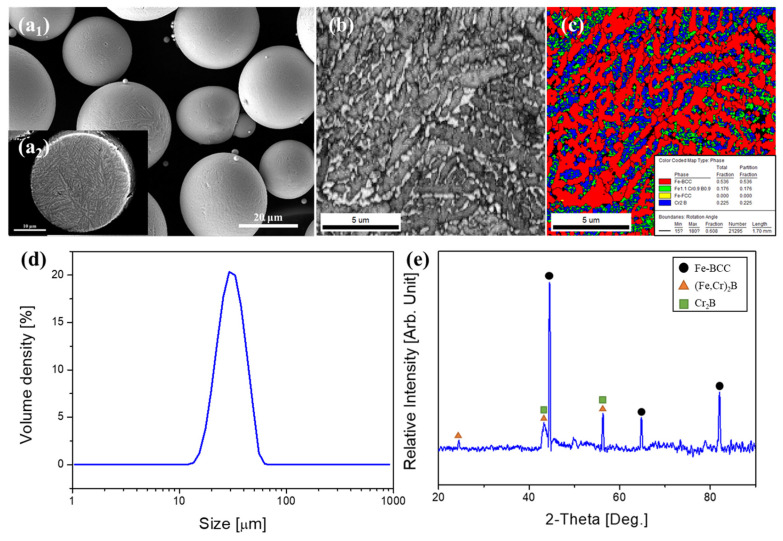
(**a1**) Morphology, (**a2**) cross-sectional image, (**b**) image quality map, (**c**) phase map, (**d**) particle size distribution, and (**e**) X-ray diffraction pattern of the as-fabricated HXA5 powders.

The morphological and microstructural characteristics of the HXA5 powder feedstock are shown in Figure 1. As presented in Figure 1(a1), the powders exhibit a predominantly spherical morphology, which is advantageous for uniform feeding during HVOF processing. The cross-sectional microstructure of the powders (Figure 1(a2)) revealed the presence of two distinct contrast regions—bright and dark—forming a well-defined dendritic structure. Electron backscatter diffraction (EBSD) analysis further identified these regions as Fe-BCC, (Cr,Fe)_2_B, and Cr_2_B phases, with corresponding volume fractions of 53.6%, 17.6%, and 22.5%, respectively (Figure 1b,c). The particle size distribution ranged from 12 µm to 55 µm, with a median diameter (D_50_) of 30.3 µm, confirming its suitability for the HVOF process (Figure 1d). The XRD analysis of the powders revealed Fe-BCC as the primary phase, accompanied by the detection of the (Cr,Fe)_2_B and Cr_2_B phases. All XRD peaks exhibited sharp and well-defined patterns, indicating the absence of a metallic glass phase in the powder feedstock (Figure 1e).

### 3.2. Characterization of HVOF Coating Materials

**Figure 2:**
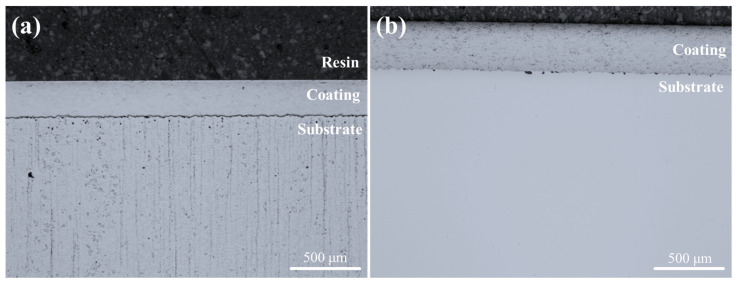
Optical micrographs of (**a**) the HXA5 coating layer and (**b**) the WC-12Co coating layer.

Figure 2 presents the cross-sectional micrographs of the fabricated coating layers. The average coating thickness values were measured as 259.6 ± 3.5 µm for the HXA5 coating and 357.4 ± 8.4 µm for the WC-12Co coating. The corresponding average porosity values were 0.78 ± 0.58% and 0.88 ± 0.13%, respectively, indicating that both coatings were successfully deposited with dense and structurally sound microstructures.

**Figure 3:**
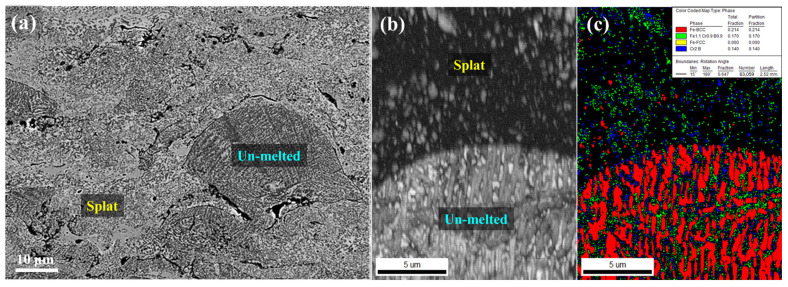
(**a**) Back scattered electron image, (**b**) image quality map, and (**c**) phase map of HXA5 coating layer.

Figure 3 illustrates the SEM and EBSD analysis results of the HVOF HXA5 coating layer. The coating exhibited two distinct areas: un-melted powder areas, in which the particle morphology was largely retained due to insufficient melting, and splat areas formed by fully molten particles deposited during spraying (Figure 3a). The un-melted powder areas largely preserved the characteristics of the initial microstructure observed in the feedstock powders (Figure 1(a2)), whereas the splat areas displayed markedly finer dark-contrast microstructures. This refinement is attributed to the rapid solidification of fully molten particles, leading to the fine re-precipitation of borides and Fe-based BCC phases. EBSD analysis also revealed that the un-melted powder areas exhibited a phase distribution similar to that of the original powders. In contrast, the splat areas showed a significant increase in areas indexed as “zero solution” (black areas in Figure 3c) due to the absence of detectable Kikuchi patterns (Figure 3b,c). According to Ham et al. [19], the lack of Kikuchi patterns in metamorphic alloys can be indicative of amorphization phenomena.

**Figure 4:**
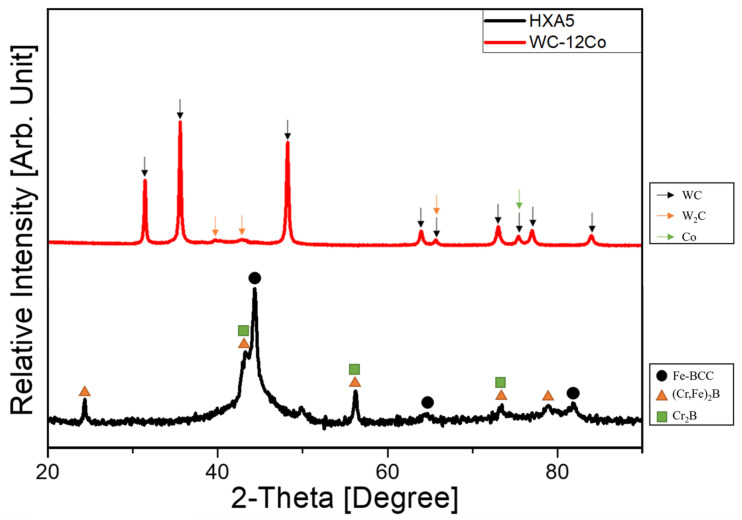
X-ray diffraction analysis results of the HXA5 and WC-12Co coating layers.

The XRD patterns of the HXA5 and WC-12Co coating materials are presented in Figure 4. For the HXA5 coating, the Fe-based BCC phase was identified as the primary diffraction peak; however, a noticeable peak broadening was observed compared with the feedstock powders. Peak broadening is primarily associated with a reduction in crystallite size, as the full width at half maximum (FWHM) of the diffraction peak increases with decreasing crystallite dimensions. This observation suggests that the rapid solidification of fully molten particles during HVOF spraying promoted the formation of nanocrystalline phases and potentially metallic glass, which is consistent with previous reports on metamorphic alloy coatings produced via thermal spray processes [12,13,18,19,20]. In contrast, the XRD analysis of the WC-12Co coating revealed WC, W_2_C, and Co as the dominant crystalline phases.

**Figure 5:**
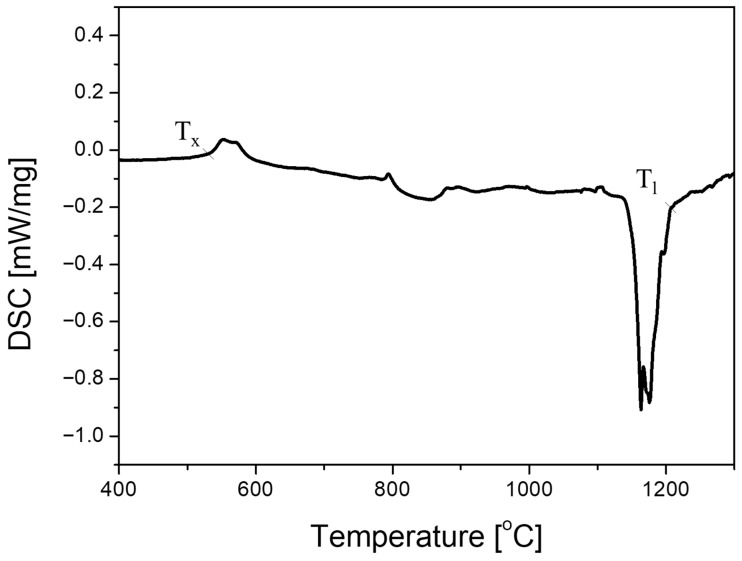
Differential scanning calorimetry curve of the HXA5 coating layer showing crystallization and liquidus temperatures.

To verify the formation of metallic glass in the HXA5 coating layer, DSC analysis was performed, and the results are presented in Figure 5. The DSC curve of the HXA5 coating exhibited an exothermic peak associated with crystallization in the temperature range of 550–600 °C, indicating the presence of metallic glass in the coating. The crystallization temperature (T_x_ was determined to be 553.3 °C, while the glass transition temperature (T_g_) could not be clearly identified. The enthalpy of crystallization was calculated to be 15.61 J/g. In our previous studies, the T_x_ of various metamorphic alloys were measured as follows: Armacor^TM^ M: 536 °C, Fe_69.1_Cr_20_B_2_C_0.4_Mo_5_Nb_3.5_: 603 °C, and Fe_70.85_Cr_20_B_2_C_0.4_Mo_5_Nb_1.75_: 579 °C [18]. Compared with these alloys, the HVOF HXA5 coating layer exhibited a T_x_ lower than that of Nb-containing metamorphic alloys but slightly higher than that of the commercial Armacor^TM^ M. This result implies that the metallic glass present in HXA5 is expected to exhibit better thermal stability than the metallic glass observed in Armacor^TM^ M. Additionally, the HXA5 coating layer showed an endothermic reaction associated with melting, starting at approximately 1152.2 °C, with the liquidus temperature (T_l_) determined to be 1172.5 °C. The enthalpy of fusion was calculated as −142.2 J/g.

**Figure 6:**
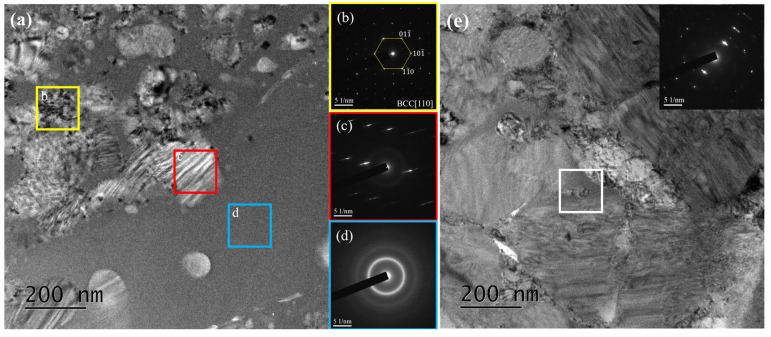
(**a**) TEM image acquired from the splat area of the HXA5 coating and selected area electron diffraction (SAED) patterns of (**b**) Fe-BCC, (**c**) borides, and (**d**) the metallic glass phase obtained from the color-boxed areas in (**a**). (**e**) TEM image acquired from the un-melted powder area and the corresponding SAED pattern obtained from the white-boxed area in (**e**). A 140 nm diameter selected area aperture was used to acquire the SAED patterns.

**Figure 7:**
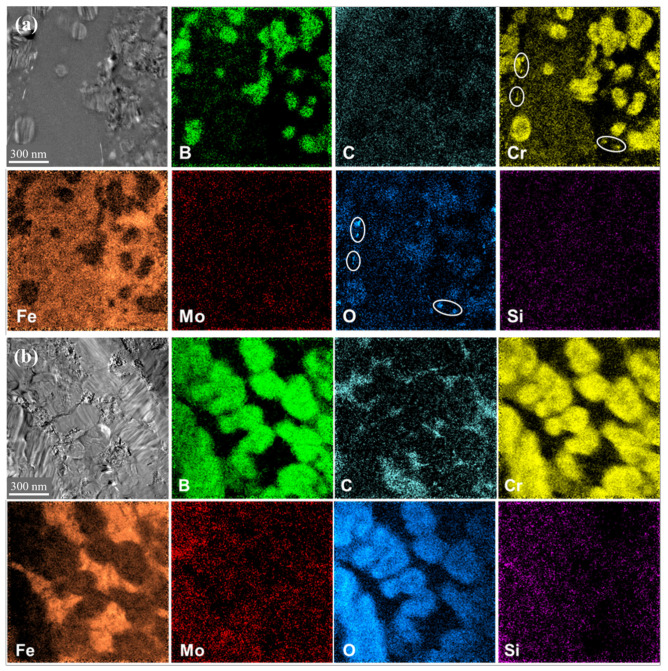
Electron energy loss spectroscopy (EELS) mapping images acquired from (**a**) the splat area and (**b**) the un-melted powder area.

To verify the presence of metallic glass and potential nanocrystalline phases in the HXA5 coating layer, TEM analysis was conducted, and the results are presented in Figure 6. In the splat area of the HXA5 coating, a characteristic halo ring pattern, indicative of metallic glass, was observed in the selected area electron diffraction (SAED) pattern (Figure 6d). This confirms that the initially fully crystalline feedstock powders underwent partial amorphization during the HVOF spraying process. In addition, crystal diffraction patterns corresponding to the Fe-based BCC phase and boride phases were also identified in the splat area (Figure 6b,c). By contrast, no metallic glass phase larger than the selected area aperture size (140 nm diameter) was detected in the un-melted powder area (Figure 6e). This indicates that metallic glass was scarcely present in this region. Figure 7 presents the EELS analysis results of the HXA5 coating layer. The Fe matrices in both the un-melted powder area and the splat area exhibited high Fe content; however, differences were observed in the concentrations of the alloying elements. Specifically, the Fe matrix in the splat area contained relatively higher amounts of B and Cr compared with that in the un-melted powder area. In contrast, the un-melted powder area exhibited elevated B and Cr contents only within the boride regions. These elements are well known to enhance the glass-forming ability (GFA) of Fe-based metallic glass alloys [24]. This observation suggests that during the HVOF process, the rapid cooling of fully molten particles promoted the partial dissolution of B and Cr into the Fe matrix, which in turn contributed to the formation of metallic glass. In general, the formation of an amorphous phase occurs when the free energy of certain crystalline phases exceeds that of the amorphous state, i.e., when the thermodynamic stabilities of the crystalline phases decrease [25]. Meanwhile, the boride phases in the splat area exhibited high intensities of B and Cr, along with minor Fe detection (Figure 7a). In contrast, the boride phases in the un-melted powder area showed high intensities of B, Cr, and O, with Fe also detected in minor amounts (Figure 7b). These observations are consistent with the EBSD and XRD analysis results presented in Figure 3 and Figure 4. In addition, trace distributions of Cr oxides were observed within the splat area (white circle in Figure 7a). The simultaneous detection of Cr and O is attributed to in-flight oxidation occurring during the HVOF spraying process. To identify the metallic glass fraction within the HXA5 coating layer, chemical etching was performed, and its microstructure was observed using optical microscopy (OM), as presented in Supplementary Figure S1. In metamorphic coatings, chemical etching selectively corrodes the less corrosion-resistant crystalline phases, which appear darkened, whereas the metallic glass phase remains bright due to its superior corrosion resistance. After chemical etching, the un-melted powder areas became noticeably darker, and some darkened regions were also observed within the splat areas. By quantifying the area fractions of the bright microstructures, corresponding to metallic glass, and the dark microstructures, corresponding to crystalline phases, the fractions were determined to be 55.6% and 44.4%, respectively, with a standard deviation of 3.7%.

### 3.3. Hardness and Wear Properties at Room Temperature

The micro-Vickers hardness test results for the HVOF coating materials are presented as follows: the HXA5 coating exhibited an average hardness of 872 ± 79.6 HV_0.3_, while the WC-12Co coating reached 1053 ± 194.2 HV_0.3_. Although the hardness of the HXA5 coating is lower than that of the representative wear-resistant WC-12Co coating, it is still considerably high compared with typical metallic materials. This elevated hardness can primarily be attributed to the strengthening contributions of the uniformly distributed boride phases and metallic glass within the HXA5 coating material. WC-based MMCs are known to exhibit the highest hardness among engineering materials, excluding diamond. Their superior hardness arises from the strong reinforcement effect of WC particles, and the hardness is inversely correlated with the particle size of WC [26,27]. In this study, the average WC particle size of the WC-12Co coating was measured to be 3.82 µm, and the corresponding microstructural observations are provided in Supplementary Figure S2.

**Figure 8:**
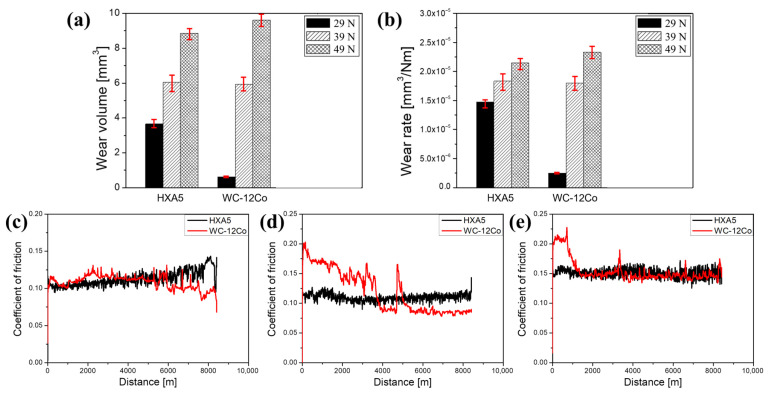
(**a**) Wear volumes, (**b**) wear rates, and coefficient of friction curves recorded during the wear tests at loads of (**c**) 29 N, (d) 39 N, and (**e**) 49 N.

Figure 8a shows the wear volume losses obtained from the wear tests. For both coating materials, the wear volume generally increased with increasing applied load. Notably, despite the hardness difference of 180.80 HV_0.3_ between the HXA5 and WC-12Co coatings, the two coatings exhibited comparable wear volumes at a 39 N load condition. And at a 49 N load condition, the HXA5 coating even demonstrated superior wear resistance (lower wear volume loss). Based on the measured wear volumes, the wear rates were calculated using Equation (1), and the results are presented in Figure 8b. The variation in wear rate with applied load followed a trend similar to that of wear volume loss. According to the theory proposed by Archard et al. [28], wear resistance is generally proportional to hardness. However, several studies have reported that wear behavior does not always correlate directly with hardness [29,30]. Leyland et al. [29] noted that while high hardness is often accompanied by a high elastic modulus, effective wear resistance also requires the ability to absorb deformation energy. Consequently, it can be inferred that the wear performance of HXA5 coating material is influenced not only by the hardness-enhancing effects of the metallic glass and boride phases but also by additional mechanisms contributing to its wear resistance. Figure 8c–e presents the variations in the coefficient of friction (COF) during the long-distance wear tests. At a 29 N load, the COF of the HXA5 coating gradually increased with sliding distance, whereas that of the WC-12Co coating fluctuated initially and then progressively decreased. At 39 N, the COF of the HXA5 coating exhibited a stable trend from the early stage of testing, while the COF of the WC-12Co coating decreased significantly before reaching a stable state. A similar trend was observed at 49 N, where the COF of HXA5 remained stable, whereas the WC-12Co coating showed an initial decrease in COF followed by stabilization. The decrease in COF suggests that certain factors act to reduce the frictional force as wear progresses. The observed decrease in COF suggests that specific mechanisms act to reduce frictional forces as wear progresses, typically associated with the formation of tribofilms during the wear process [31,32]. This phenomenon will be discussed in more detail in Section 4.2. Furthermore, the COF curves of the WC-12Co coating exhibited more frequent spikes compared with those of the HXA5 coating. These spikes are attributed to the crushing of WC particles and the subsequent material delamination, as elaborated in the following chapter. The stable COF behavior observed over long sliding distances indicates favorable tribological stability, which can be advantageous for the long-term service performance of the coating materials.

**Figure 9:**
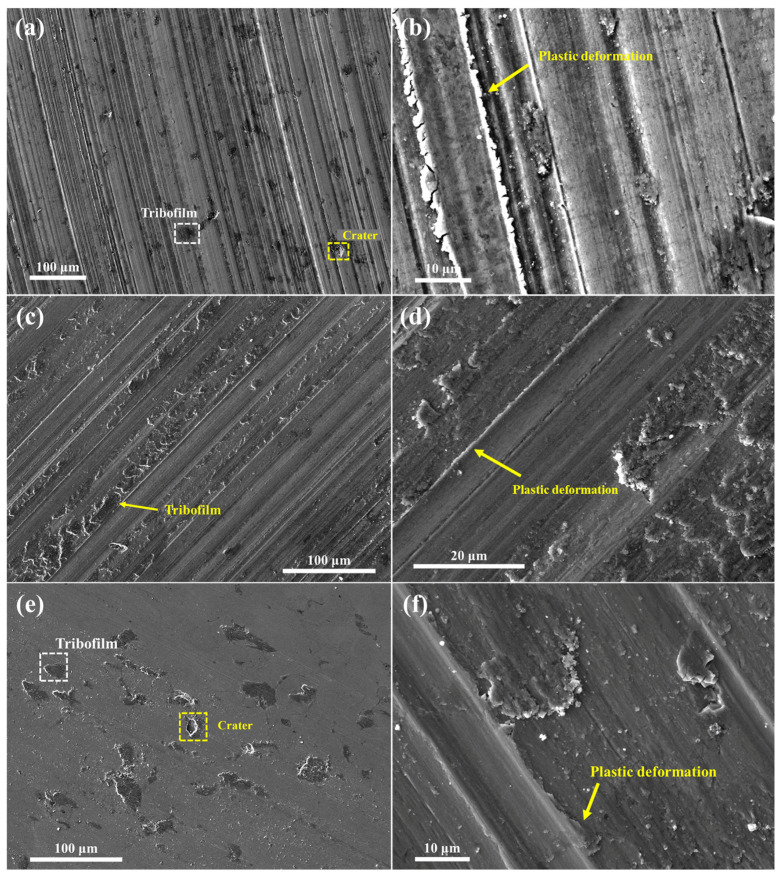
Surface images of HXA5 coating after the wear tests at loads of (**a**,**b**) 29 N, (**c**,**d**) 39 N, and (**e**,**f**) 49 N.

**Figure 10:**
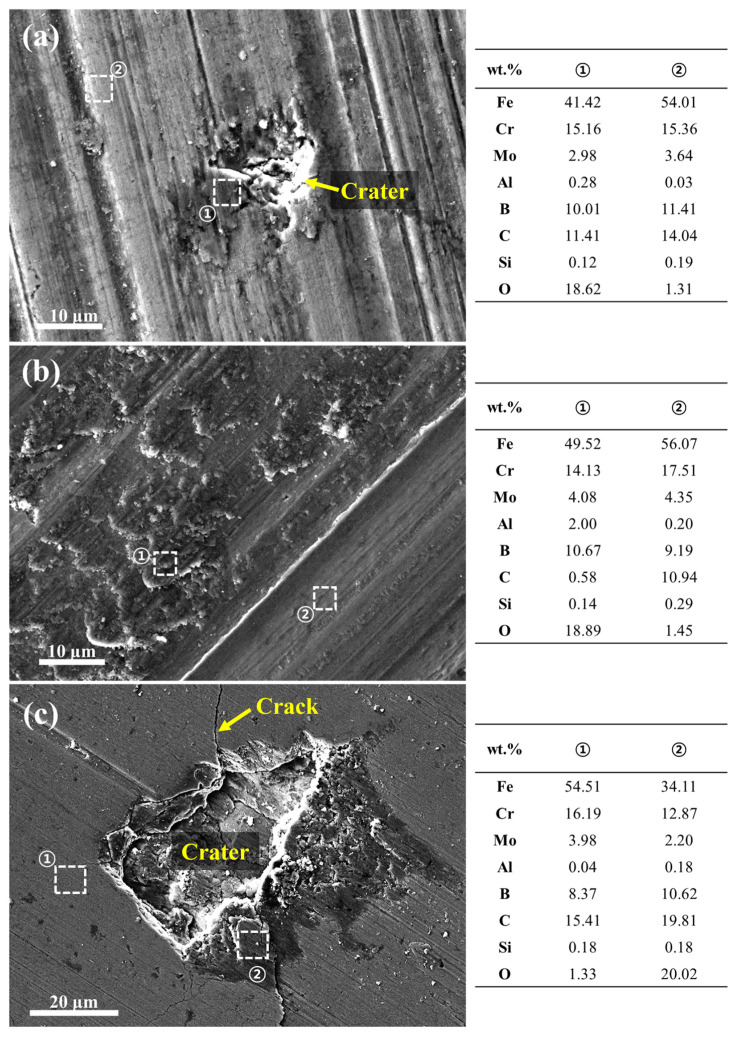
SEM/EDS analysis results of the HXA5 coating surfaces after wear tests at loads of (**a**) 29 N, (**b**) 39 N, and (**c**) 49 N.

Figure 9 presents the SEM images of the worn surfaces of the HXA5 coating after the wear tests. Across all tested conditions, clear grinding marks were observed, indicating that the dominant wear mechanism of this material is abrasive wear. In addition, dark tribofilms were formed along the wear tracks, and localized plastic deformation was observed at the edges of the grinding marks. To identify the elemental composition of the tribofilms, an additional EDS analysis was conducted, and the results are shown in Figure 10. The tribofilm regions exhibited significantly higher oxygen contents compared with the surrounding areas, suggesting that the tribofilms were generated through oxidation reactions induced by flash temperatures during the friction wear process. In certain regions of the worn surface, craters were observed, which appeared to have formed due to the delamination of the tribofilm or coating material (Figure 9a,e and Figure 10a,c). Under the 49 N load condition, cracks were also detected around the craters, which are presumed to have originated from the fracture of particle boundaries weakened by in-flight oxidation [2,18].

**Figure 11:**
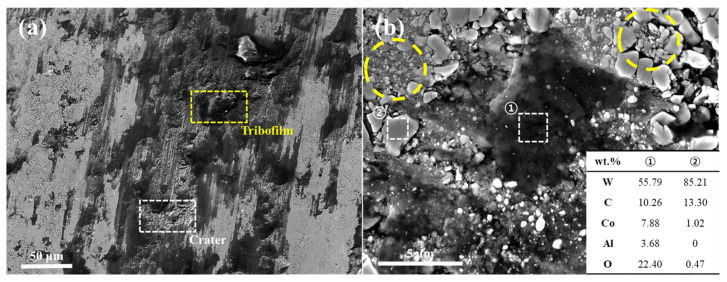
(**a**) Low-magnification and (**b**) high-magnification images of the worn surface of the WC-12Co coating after the wear test at a load of 49 N.

Meanwhile, Figure 11 presents the worn surface images of the WC-12Co coating tested under the most severe load condition of 49 N. Similarly to the HXA5 coating, the worn surfaces of WC-12Co exhibited the formation of tribofilms and craters (Figure 11a). To identify the elemental composition of the tribofilms, EDS analysis was performed, and the results are shown in Figure 11b. The tribofilm regions exhibited high intensities of Co, which serves as the binder phase, along with elevated oxygen contents. Based on these observations, it can be inferred that the binder phase was preferentially removed during wear, followed by oxidation reactions that led to the formation of the tribofilm. This phenomenon is consistent with the wear behavior of WC-based MMCs reported by Ghosh et al. [33]. In addition, Figure 11b shows that some WC particles became more irregular in shape and finer in size compared with those observed in Figure S2 (highlighted with yellow dashed circles in Figure 11b). This indicates particle crushing under high-load conditions, a phenomenon also reported by Wang et al. in WC–Co–Cr coatings [34]. Such WC particle crushing can induce three-body wear and accelerate crater formation through particle delamination. This mechanism is also closely associated with the COF spikes observed in Figure 8, suggesting a direct link between particle fracture, material delamination, and friction instability under severe wear conditions.

## 4. Discussion

### 4.1. Amorphization of Metamorphic Alloy During the HVOF Process

Metallic glass can be formed by suppressing the nucleation of crystalline phases through rapid quenching from the liquid state. In relation to this, three empirical rules have been proposed to enhance GFA: (1) the system should be a multicomponent alloy with at least three principal elements, (2) the atomic size mismatch among the three main constituents should exceed 12%, and (3) the mixing enthalpy between the principal elements should be negative [35]. Miracle et al. [36], building upon the topology model of Egami et al. [37], proposed a modified model for the case in which solute atoms occupy interstitial sites, demonstrating that GFA is most strongly influenced by atomic size mismatch. The atomic radii of the constituent elements in the HXA5 alloy are as follows [38]: Fe (1.40 Å), Cr (1.40 Å), Mo (1.45 Å), B (0.85 Å), C (0.70 Å), and Si (1.10 Å). Cr and Mo, as substitutional elements, occupy lattice sites; however, their atomic radii are similar to that of Fe, suggesting that the effective enhancement of GFA requires the addition of metalloid elements such as C, Si, B, P, S, or Be [39]. Wu et al. [40] reported that, in the Fe-Cr system, the addition of C is more effective in enhancing GFA than B or P. The Fe-C system can provide the highest driving force for metallic glass formation and the greatest resistance to crystallization. In addition, Inoue et al. [41] demonstrated that introducing 1.5 at.% B into the Fe-C-Si system reduced the cooling-rate sensitivity required for amorphous formation, enabling the fabrication of metallic glass with a thickness of up to 500 µm. These findings indicate that the combined addition of C and B can effectively improve GFA by delaying the nucleation of the α-Fe phase [42,43,44]. The GFA of a material can be evaluated using the reduced glass transition temperature T_rg_ (=T_g_/T_l_), which can be derived from DSC analysis [45]. In the case of HXA5, T_g_ was not clearly distinguishable in Figure 5; therefore, T_x_ was assumed to be approximately equal to T_g_ for the calculation, yielding a T_rg_ value of 0.472. For comparison, the T_rg_ of Fe_41_Cr_15_Mo_14_C_15_B_6_Y_2_ metallic glass [46] has been reported as 0.573, suggesting that the GFA of HXA5 is lower than that of typical Fe-based metallic glass alloys. This is consistent with the partial presence of the Fe-based BCC phase observed in the splat areas of the HVOF HXA5 coating (Figure 6b). Nevertheless, the HXA5 alloy contains more than 20 at.% B, which dissolves during the HVOF spraying process and suppresses the nucleation of crystalline Fe-based BCC phases during rapid solidification (Figure 7a). Consequently, despite its lower GFA relative to conventional Fe-based metallic glasses, a significant fraction of metallic glass was still formed within the splat areas (Figure 6d).

### 4.2. Wear Behavior of Metamorphic Alloy at Room Temperature

The wear resistance of the HVOF HXA5 coating layer was lower or comparable to that of the representative wear-resistant WC-12Co coating at low to intermediate wear loads but exhibited superior wear performance at higher load conditions (Figure 8). The outstanding wear resistance of WC-based MMCs primarily originates from the high hardness of WC particles and the lubricating effect provided by the tribofilms formed after the extrusion of the softer Co binder phase. Although the average hardness value of the HXA5 coating is lower than that of WC-12Co, the hardness difference between the reinforcing boride and the metallic matrix in HXA5 is considerably smaller compared with the hardness difference between WC particles and the Co binder. Specifically, the hardness of WC particles ranges from approximately 23.7 to 44.3 GPa, whereas the Co binder exhibits a significantly lower hardness between 1.4 and 7.6 GPa [47,48]. In contrast, the (Cr,Fe)_2_B boride in the HXA5 alloy has a reported hardness of approximately 27 GPa [49], and the Fe-Cr-B metallic glass matrix possesses hardness values ranging from 18.37 to 21.86 GPa [2]. As previously mentioned, the reduced hardness mismatch between the reinforcing boride and the metallic glass matrix in HXA5 is expected to significantly mitigate the detachment of reinforcing phases under high-load wear conditions, thereby contributing to the enhanced wear resistance observed.

Leyland et al. [29] reported that the wear behavior of materials is influenced not only by hardness but also by their ability to absorb deformation energy. Metallic glasses, in general, exhibit high strength due to the absence of conventional defects such as dislocations and grain boundaries; however, they have limited capacities for plastic deformation. During sliding wear tests, continuous sliding can generate frictional heat. Several studies have reported that under such combined stress and temperature conditions, viscous flow can be induced in metallic glasses [35,50,51,52]. Once viscous flow occurs, plastic deformation can take place even under stresses below the intrinsic yield strength of the material [53]. In the HXA5 coating layer, evidence of plastic deformation during wear was observed, as shown in Figure 8. In contrast, the WC-12Co coating exhibited almost no signs of plastic deformation beyond tribofilm formation and WC particle detachment. This behavior can be attributed to the microstructural characteristics of WC-based MMCs, in which the fraction of rigid WC particles exceeds 80%, leaving the Co binder insufficient to accommodate significant plastic deformation.

In addition, the tribofilm formed during the wear test can also influence the wear resistance. According to Iwabuchi et al. [31,32], when oxides generated during the wear process form a tribofilm, the oxides act as solid lubricants, leading to a gradual decrease in the coefficient of friction (COF). This phenomenon is considered to be closely related to the sharp drop in COF observed in the WC-12Co coating layer shown in Figure 7. In contrast, the HXA5 coating layer did not exhibit a rapid COF decrease but rather maintained a consistently low COF from the beginning to the end of the test. This behavior is attributed to the significantly higher proportion of the Fe-based matrix in the HXA5 coating compared with the Co content in the WC-12Co coating, enabling the HXA5 coating material to rapidly form a tribofilm immediately upon the onset of friction. The ability of the HXA5 coating to maintain stable and consistent COF values may be linked to the absence of extensive boride particle crushing. As shown in Figure 10, the WC-12Co coating tested at 49 N exhibited fractured and fragmented WC particles, consistent with the findings of Wang et al. for WC–Co composites under high-load wear conditions [34]. Under such conditions, the crushing and cracking of WC particles, coupled with plastic deformation of the binder, delay the stabilization of COF and accelerate wear damage. Detached WC fragments and craters formed by particle delamination further introduce irregularities in the wear response, as reflected in the COF spikes observed in Figure 8. By contrast, although the HXA5 coating also exhibited localized cracking along some particle boundaries and crater formation due to tribofilm delamination, the extent of these damage modes appeared less severe than in WC-12Co. This relative suppression of particle fragmentation and delamination is considered a key factor contributing to the more stable tribological response of the HXA5 coating under severe wear conditions.

In summary, although the HXA5 coating exhibits lower hardness than the WC-12Co coating, its superior wear resistance can be attributed to the combined effects of (1) the relatively small hardness difference between the Fe-based matrix and the boride; (2) the plastic deformation accommodation of the metallic glass; and (3) the solid lubrication effect of the tribofilm.

## 5. Conclusions

In this study, a newly designed Fe-based metamorphic alloy (HXA5) coating layer was fabricated using the high-velocity oxygen fuel (HVOF) thermal spray process. Microstructural characterization and room-temperature wear tests were conducted, and the following conclusions were drawn:

1. The HXA5 coating layer fabricated via the HVOF process was successfully manufactured without the formation of significant cracks or porosity. The internal microstructure consisted of distinct splat areas and un-melted powder areas. In the splat areas, the microstructure was composed of metallic glass and (Cr,Fe)_2_B phases, with some residual Fe-based BCC phase present. The un-melted powder areas exhibited a microstructure similar to that of the initial powders, consisting of a BCC matrix and dendrite-like borides.
2. Room-temperature pin-on-disk wear tests revealed that the HXA5 coating exhibited wear resistance comparable to that of the WC-12Co coating, and at high-load condition, it even demonstrated superior wear resistance. The excellent wear performance of the HVOF HXA5 coating is attributed to the small hardness mismatch between the metallic glass and boride phase within the Fe-based matrix, the ability of the metallic glass to accommodate plastic deformation during friction wear, and the formation of oxide tribofilms that act as solid lubricants.
3. In summary, the newly developed Fe-based metamorphic alloy coating layer exhibited wear resistance comparable to that of the WC-12Co metal matrix composite, demonstrating significant industrial potential and suggesting that it could meaningfully contribute to extending the service life of various components in the future.

## Figures and Tables

**Table 1:** Chemical composition of the HXA5 powders used in this study.

	Fe	Cr	Mo	Mn	B	Si	C
wt.%	Bal.	23	6	0.5	5	1.5	1
at.%	Bal.	19.74	2.79	0.41	20.64	2.38	3.71

**Table 2:** HVOF process parameters for the fabrication of the HXA5 coating.

Fuel	Fuel Flow [m^3^/h]	Oxygen Gas Flow [m^3^/h]	Carrier Gas	Carrier Gas Flow [m^3^/h]	Feeding Rate [g/min]	Distance [mm]	Barrel [mm]
Kerosene	1.82	50.97	Nitrogen	0.88	50	350	101.6

## Data Availability

The original contributions presented in this study are included in the article/Supplementary Materials. Further inquiries can be directed to the corresponding author.

## Supplementary Materials

The following supporting information can be downloaded at: https://www.mdpi.com/article/10.3390/ma18184241/s1, Figure S1: Optical micrograph of the HXA5 coating layer after severe chemical etching; Figure S2: Initial microstructure of WC-12Co coating layer.
