# Supplementary material for: Fe-Cr-Mo-B-Si-C Metamorphic Alloy Coating with Excellent Wear Resistance Fabricated via High-Velocity Oxygen Fuel Thermal Spray Process
Source: Materials (Basel). 2025 Sep 10;18(18):4241. doi: 10.3390/ma18184241 (PMC12471290; doi:10.3390/ma18184241)
Supplement: Supplementary file 1 [file materials-18-04241-s001.zip › materials-3840840-supplementary.pdf]

Supplementary Materials

Fe-Cr-Mo-B-Si-C Metamorphic Alloy Coating with Excellent Wear Resistance Fabricated Via High- Velocity Oxygen Fuel Thermal Spray Process

Yu-Jin Hwang[1], Yong-Hoon Cho[1], Gi-Su Ham[2], Choongnyun Paul Kim[2], and Kee-Ahn Lee[1*]

[1] *Department of Materials Science and Engineering, Inha University, Incheon, 22212, Republic of Korea*

[2] *KOLON Advanced Research Cluster, Kolon Industries Inc., Seoul 07793, Republic of Korea*

[*] Corresponding author. Department of Materials Science and Engineering, Inha University, Incheon 22212, Republic of Korea. E-mail address: keeahn@inha.ac.kr (Kee-Ahn Lee)

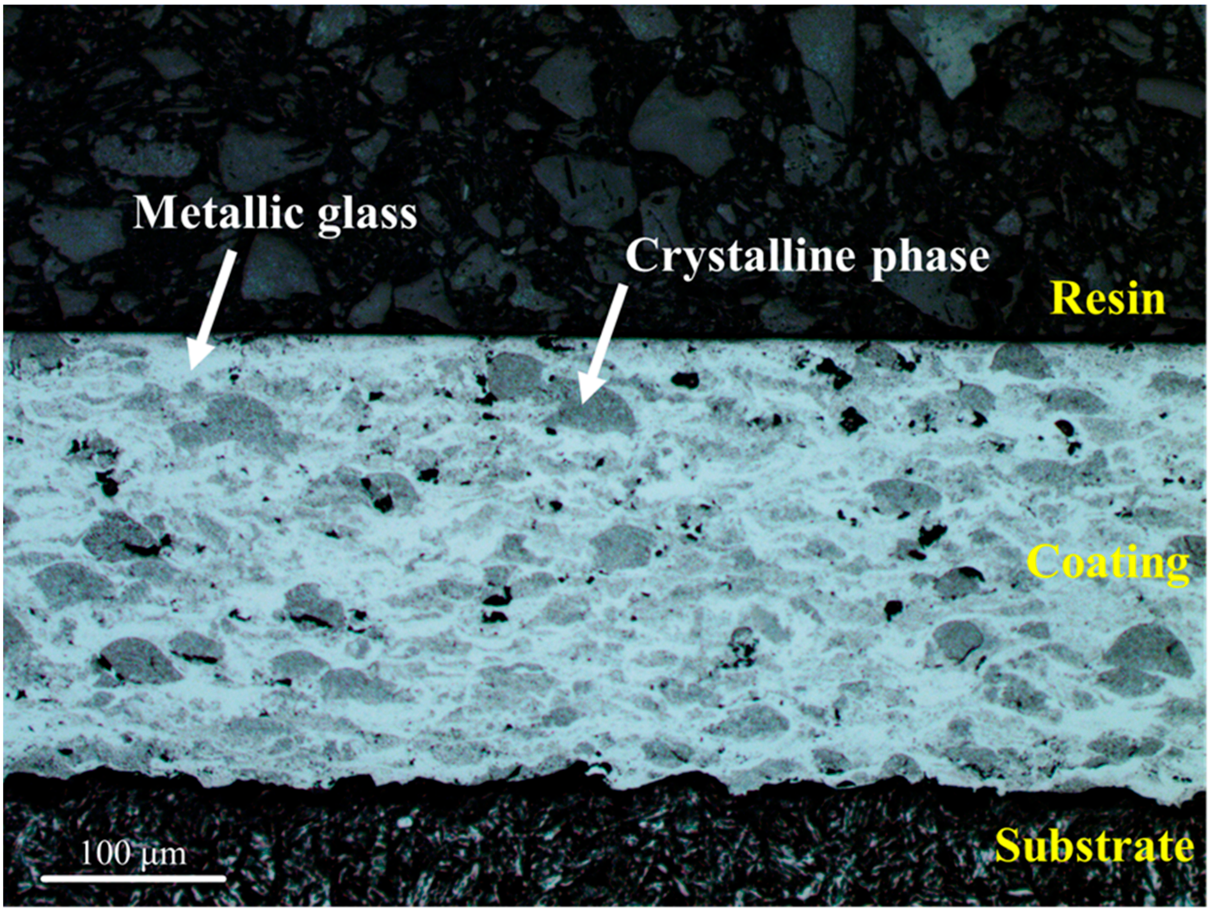

Figure S1. Optical micrograph of the HXA5 coating layer after severe chemical etching.

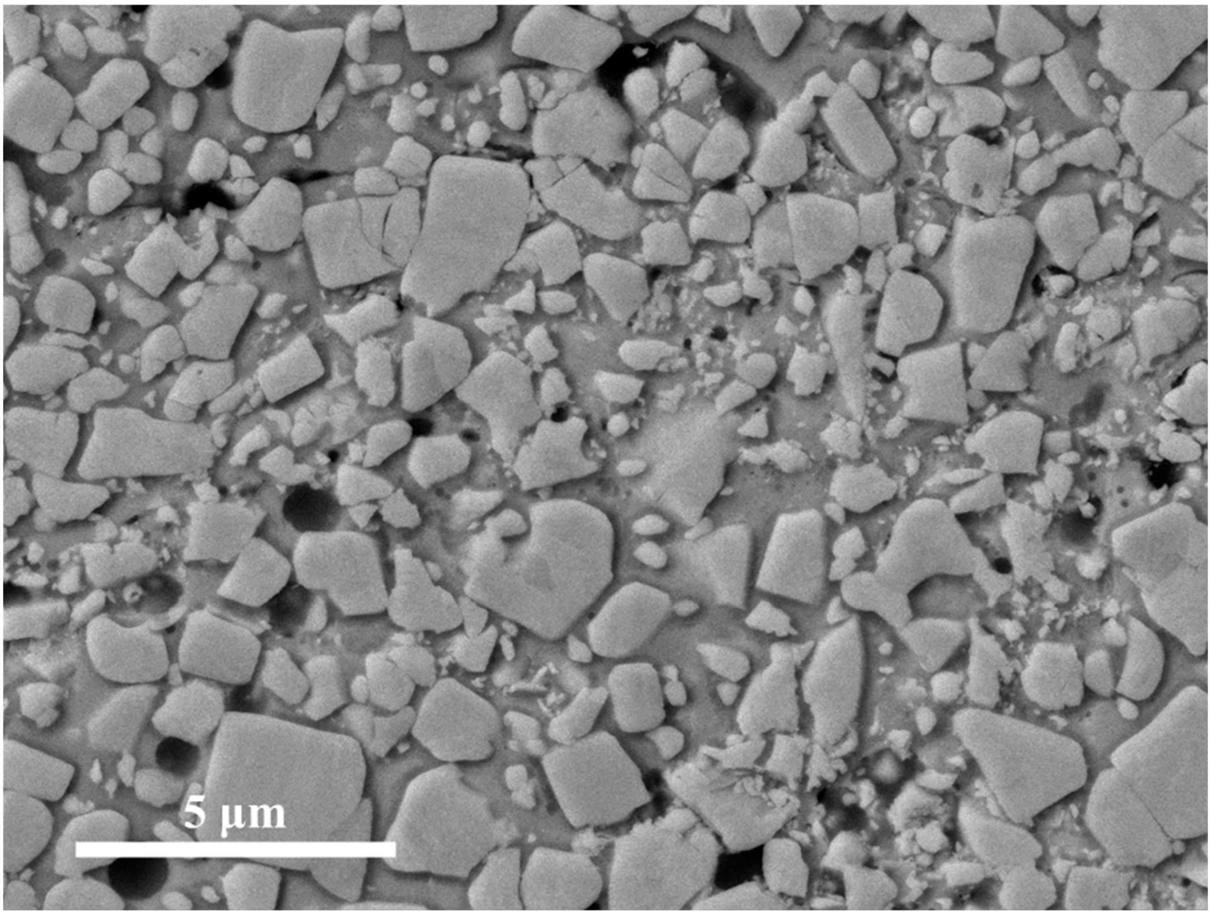

Figure S2. Initial microstructure of WC-12Co coating layer.
